# Regulation of apoptosis and interaction with cartilage degeneration in osteoarthritis

**DOI:** 10.3389/fcell.2025.1571448

**Published:** 2025-03-27

**Authors:** Jiahua Mei, Niqin Xiao, Yujiang Xi, Xin Chen, Xuezhi Zha, Lili Cui, Fei Yan, Rui Xue, Yongsen Wang, Yunshu Ma

**Affiliations:** ^1^ Yunnan University of Chinese Medicine, Kunming, China; ^2^ The Key Laboratory of External Drug Delivery System and Preparation Technology in University of Yunnan Province, Kunming, China; ^3^ Yunnan Key Laboratory of Dai and Yi medicines, Kunming, China

**Keywords:** osteoarthritis, apoptosis, cartilage degeneration, signaling pathways, molecular mechanisms

## Abstract

Osteoarthritis (OA) is a chronic degenerative joint disease, primarily characterized by the degradation of the ECM and cartilage degeneration. Articular cartilage is maintained by chondrocytes, which secrete the ECM, making the stability of these cells crucial for joint function. Research has shown that in the later stages of OA, cartilage cavities form, indicating a decline in chondrocyte function. Chondrocyte death is considered a central feature of cartilage degeneration. Apoptosis, a form of programmed cell death, plays a key role in this process. While controlled apoptosis helps remove damaged chondrocytes and protects the cartilage from injury, excessive apoptosis disrupts the balance of the cartilage microenvironment and accelerates OA progression. Therefore, regulating chondrocyte apoptosis may offer a novel approach for preventing and treating cartilage degeneration. This review examines the apoptosis pathways, the interaction between apoptosis and OA, the key regulatory factors of chondrocyte apoptosis, and analyzes current drug interventions targeting apoptosis in both preclinical and clinical studies. It also discusses the challenges in treating OA and outlines future research directions to guide upcoming studies.

## 1 Introduction

OA is the most common chronic joint disease worldwide, characterized by cartilage degeneration, osteophyte formation, synovial hyperplasia, and other related features (Hayashi et al., 2025; Mündermann et al., 2024). OA has a high prevalence, with the majority of individuals over the age of 65 worldwide affected by this condition. It typically presents with symptoms such as joint pain, stiffness, swelling, and deformity, significantly impacting patients’ quality of life (Minnig et al., 2024). Although the exact cause of OA remains unclear, it is generally attributed to factors such as aging, obesity, genetics, cumulative joint wear, and joint injuries. Among these, age is considered the most significant risk factor. As the global population ages, the number of OA patients is expected to increase annually (Segal et al., 2024). Current treatment options for OA mainly include nonsteroidal anti-inflammatory drugs, corticosteroids, and chondroprotective agents, which can alleviate pain to some extent but are associated with significant side effects. For advanced OA, joint replacement surgery is often considered; however, challenges such as limited joint reserve and durability persist, and these treatments cannot effectively halt the progression of OA (Courties et al., 2024). Therefore, understanding the pathogenesis and exploring early prevention strategies for OA are critical, as is the development of safe and effective emerging therapies.

Data show that OA patients experience varying degrees of cartilage degeneration (Gilbert et al., 2024). Articular cartilage consists of the superficial, intermediate, and deep zones, with key functions including providing a smooth surface, reducing friction between bones, alleviating local pressure, maintaining joint morphology, and facilitating joint movement (Arai et al., 2024). Chondrocytes are the only cell type in cartilage tissue and play a vital role in maintaining cartilage homeostasis by producing the extracellular matrix (ECM). Preserving cartilage homeostasis is crucial for preventing and delaying OA (Makarczyk, 2023). Increasing evidence suggests that the reduction in chondrocyte numbers is a major cause of cartilage degeneration. The loss of chondrocytes in joint cartilage is often linked to apoptosis, which directly or indirectly contributes to cartilage degeneration (Kong et al., 2024).

Apoptosis, a specific form of programmed cell death, is one of the earliest forms of cell death identified (Newton et al., 2024). In normal articular cartilage, apoptosis occurs at low levels to remove damaged cells and protect cartilage from injury. However, in the later stages of OA, the number of apoptotic cells increases, leading to inflammatory responses that exacerbate cartilage degeneration (Lee et al., 2024). Therefore, regulating chondrocyte apoptosis may offer a feasible and effective strategy for slowing cartilage degeneration. This review discusses the pathways of apoptosis, its interaction with OA, the key regulatory factors of chondrocyte apoptosis, and analyzes drug interventions targeting apoptosis in both preclinical and clinical studies. Additionally, it addresses challenges in OA treatment and outlines future research directions to guide subsequent studies.

## 2 Pathways of apoptosis

**FIGURE 1:**
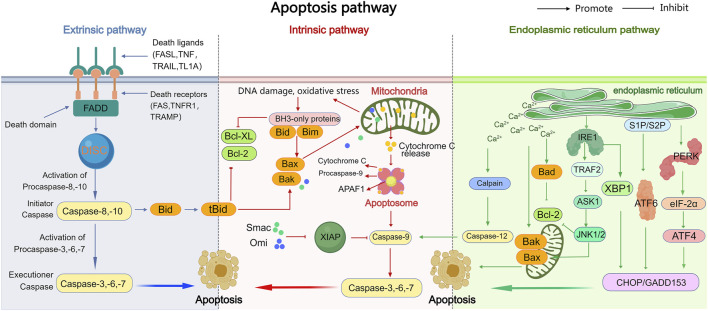
The exogenous, endogenous, and endoplasmic reticulum stress apoptotic pathways. The intrinsic, extrinsic, and endoplasmic reticulum stress pathways are the three major pathways that trigger apoptosis (programmed cell death). In the extrinsic pathway, death receptors on the cell surface bind to external signals, including FasL, TRAIL, TNF-α, and TL-1A, initiating a series of events that ultimately lead to cell death. The intrinsic pathway leads to the release of apoptotic molecules, which activate caspases and result in cell death due to mitochondrial outer membrane permeability caused by stimuli such as DNA damage or oxidative stress. Both of these pathways ultimately activate caspases, leading to various subsequent events of cell death. The endoplasmic reticulum stress pathway, while activating caspases, also activates IRE1, ATF6, and PERK, leading to subsequent cell apoptosis.

Apoptosis is a complex physiological process involving three pathways: the exogenous pathway (or death receptor pathway), the endogenous pathway (or mitochondrial pathway), and the endoplasmic reticulum (ER) stress pathway. A mechanism diagram is shown in Figure 1.

### 2.1 Death receptor pathway

Death receptors are part of the TNF receptor superfamily (TNFR superfamily) and have a structure that includes extracellular cysteine-rich domains and intracellular death domains (DD). Examples of death receptors include Fas, TNFR1, TRAILR1, and TRAMP (Zheng et al., 2023). In the exogenous apoptotic pathway, death receptors bind to specific ligands, such as FasL, TNF, TRAIL, and TL1A. Once activated, the receptors interact with corresponding death domain proteins, like FADD, which exposes a death fold known as the effector domain (DED). This interaction recruits caspase-8/10, forming the death-inducing signaling complex (DISC) (Green, 2022a). Caspase-8 then undergoes oligomerization and is activated through autocatalysis. The activated caspase-8 subsequently activates downstream effector caspases, including caspase-3/6/7. These caspases degrade various cellular structural components. For instance, caspase-3 degrades poly ADP ribose polymerase (PARP-1), the DNA fragmentation inhibitor, and gelsolin, while caspase-6 degrades the nuclear structural protein Lamin A/C, ultimately leading to apoptosis (Errami et al., 2013; Mehl et al., 2022; Valldorf et al., 2016; Yang, 2015). However, if caspase-8 is inhibited or insufficient to activate downstream caspases, the cleaved Bid (tBid) translocates to the mitochondria, activating the mitochondrial apoptotic pathway (Green, 2022b; Green, 2022c).

### 2.2 Mitochondrial pathway

The activation of the mitochondrial apoptosis pathway is triggered by internal cellular stress or damage, such as oxidative stress and DNA damage. A key event in this pathway is the increased mitochondrial outer membrane permeability (MOMP), which leads to the release of intermembrane proteins into the cytoplasm, initiating downstream signaling cascades that result in apoptosis (Kalkavan and Green, 2018). Bcl-2 family proteins primarily regulate apoptosis by controlling MOMP. When pro-apoptotic proteins like Bax and Bad are activated, they insert into the mitochondrial outer membrane, forming pores that allow macromolecules to pass through, thereby promoting MOMP(Wei et al., 2001; Chittenden, et al., 1995; Peña-Blanco and García-Sáez, 2018). The regulation of another pro-apoptotic protein, Bok, differs significantly. Bok is primarily expressed in the endoplasmic reticulum, and when the ER’s degradation capacity is overwhelmed, Bok accumulates and translocates to the mitochondria, influencing MOMP(Shalaby et al., 2020). Anti-apoptotic proteins counteract MOMP, thereby blocking the mitochondrial apoptosis pathway. When Bax and Bad are activated, they expose their BH3 domains, which open a groove. These BH3 domains can oligomerize and form dimers (Birkinshaw and Czabotar, 2017). The anti-apoptotic protein Bcl-2 binds to the exposed BH3 domain, preventing oligomerization and blocking the Bax-Bax or Bad-Bad interactions necessary for MOMP (Tait and Green, 2010; Suhaili et al., 2017). However, the strong binding affinity of the BH3 domain in BH3-only proteins allows them to still interact with the BH groove of anti-apoptotic proteins, even in the presence of both pro-apoptotic and anti-apoptotic proteins. This disrupts the anti-apoptotic proteins’ ability to interact with other proteins, thereby promoting apoptosis (Giam et al., 2008). Moreover, the BH3 domains of the BH3-only proteins Bid and Bim not only activate Bax and Bad but also trigger their oligomerization, increasing MOMP and further inducing apoptosis (Datta et al., 2002).

Increased MOMP triggers apoptosis by releasing various proteins. One of these is cytochrome C, a protein encoded by nuclear DNA, synthesized in the cytoplasm, and then transported into the intermembrane space of the mitochondrial inner and outer membranes (Dai et al., 2023). Once released, cytochrome C binds to APAF1, forming the apoptosome with caspase-9, which subsequently activates caspases-3, -6, and -7. These activated caspases initiate the degradation of various cellular substrates, ultimately leading to apoptosis (Shakeri et al., 2017; Li et al., 1997). The X-linked inhibitor of apoptosis protein (XIAP) is the most potent inhibitor of apoptosis in the IAP family. When cytochrome C triggers the formation of the APAF1 apoptosome, XIAP can prevent caspase activation and block apoptosis (Angel-Lerma et al., 2025). Another key protein released by the mitochondria is second mitochondria-derived activator of caspases (Smac), which, like cytochrome C, is produced by nuclear genes and transported into the mitochondrial intermembrane space (Du et al., 2000). Smac promotes apoptosis by binding to XIAP, preventing its inhibition of caspases and thus allowing caspase activity to proceed freely. Additionally, mitochondrial serine protease, another protein released from the mitochondria, also inhibits XIAP, further promoting apoptosis (Hegde et al., 2002).

### 2.3 ER stress pathway

ER stress is a cellular response triggered by the accumulation of unfolded or misfolded proteins in the ER lumen and disruptions in Ca^2+^ homeostasis. When cells remain under prolonged stress, the ER transmembrane proteins—such as protein kinase R-like ER kinase (PERK), inositol-requiring enzyme-1 (IRE1), and activating transcription factor 6 (ATF6)—induce apoptosis (Kapuy, 2024). Under normal conditions, proteins are properly folded, and PERK and IRE1 form stable complexes with molecular chaperones like BiP. However, when proteins fail to fold correctly, they compete with these chaperones for binding. This competition causes PERK to be released and activated, leading to the phosphorylation of the alpha subunit of the translation initiation factor eukaryotic translation initiation factor 2 alpha (eIF-2α). Phosphorylated eIF-2α activates the transcription factor ATF4, which promotes the expression of apoptotic signal molecules such as C/EBP-homologous protein (CHOP) and growth arrest and DNA damage-inducible 153 (GADD153), ultimately driving apoptosis (Hetz et al., 2020).Similarly, activated IRE1 recruits the cytoplasmic protein receptor-associated factor-2 (TRAF-2), which further activates c-Jun N-terminal kinase (JNK). The activation of JNK inhibits the anti-apoptotic proteins of the Bcl-2 family by phosphorylation, promoting apoptosis. Additionally, TRAF-2 activation leads to the activation of caspase-12, which initiates the caspase cascade and further contributes to apoptosis. IRE1 also promotes the expression of CHOP/GADD153, enhancing apoptotic processes (Huang et al., 2022; Iurlaro and Muñoz-Pinedo, 2016). Activated ATF6, in response to ER stress, is translocated to the Golgi apparatus in vesicle form before moving to the nucleus, where it induces the expression of CHOP/GADD153, thereby further promoting apoptosis (Ong et al., 2024; Hillary and FitzGerald, 2018). Additionally, ER stress disrupts Ca^2+^ homeostasis, leading to an influx of Ca^2+^ into both the cytoplasm and mitochondria. This disturbance not only alters the activity of Bcl-2 family proteins but also activates the intracellular neutral cysteine protease Calpain. Calpain, in turn, influences apoptosis by triggering the caspase cascade (Patergnani et al., 2020).

## 3 The mechanism of chondrocyte apoptosis in OA

Studies have shown that there is an increased number of apoptotic chondrocytes in the articular cartilage of OA patients. Among the various factors contributing to OA, age is considered one of the key risk factors. Research indicates that in the elderly population, the number of chondrocytes in the joints gradually decreases, and there is a strong positive correlation between cartilage damage and chondrocyte apoptosis (Zamli and Sharif, 2011). The calcification of OA cartilage in the elderly is associated with the upregulation of extracellular inorganic pyrophosphate and nucleoside triphosphate pyrophosphohydrolase (NTPPPH) levels in chondrocytes. When the NTPPPH isoenzyme is transfected *in vitro*, the degree of apoptosis in meniscal chondrocytes significantly increases, indicating a direct link between aging, apoptosis, and cartilage degeneration (Johnson et al., 2001). Furthermore, apoptotic bodies isolated from nitric oxide-cultured chondrocytes contained NTPPPH and precipitated calcium, suggesting that chondrocyte apoptosis may directly influence cartilage calcification in OA (Hashimoto et al., 1998). Additionally, age-related mitochondrial dysfunction induces oxidative stress, which is characterized by excessive accumulation of reactive oxygen species (ROS) and energy metabolism imbalance in articular chondrocytes. This oxidative stress is believed to promote chondrocyte apoptosis (Hui et al., 2016; Blanco et al., 2011; Scott et al., 2010). Therefore, aging may accelerate chondrocyte death by promoting apoptosis, thereby accelerating cartilage degeneration.

Under normal conditions, chondrocytes maintain a balance between the synthesis and degradation of the ECM. However, chondrocyte death is closely associated with ECM degradation and calcification (Hosseinzadeh et al., 2016). In the early stages of OA, inflammation and oxidative stress dominate, with apoptosis playing a dual role. On one hand, moderate apoptosis helps remove dysfunctional chondrocytes, reducing the secretion of pro-inflammatory factors and slowing cartilage degeneration. On the other hand, excessive apoptosis leads to increased production of pro-inflammatory factors, which in turn promotes chondrocytes to secrete proteases, such as matrix metalloproteinases (MMPs) and ‘aggrecanase’ a disintegrin and metalloproteinase with thrombospondin motifs (ADAMTSs). These proteases degrade the ECM and aggrecan (Lin and Liu, 2010; Yang et al., 2017), disrupting the homeostatic environment, shifting the balance toward catabolism, and further inducing chondrocyte death (Wang et al., 2011; Lee et al., 2013). Excessive chondrocyte apoptosis and ECM degradation thus accelerate cartilage degeneration. The reduced ability of chondrocytes to secrete ECM hampers joint cartilage repair, while continued cartilage damage diminishes its protective function, further worsening the severity of OA. This suggests that apoptosis plays a dual role in the early stages of OA (Yan et al., 2016).

In the later stages of OA, the primary feature is the destruction of cartilage structure and repair failure. The calcified cartilage layer, a main component of the cartilage matrix, is severely compromised. At this stage, the number of chondrocytes decreases even further, with many cavities and voids appearing, signaling a decline in the chondrocytes’ ability to secrete ECM. This exacerbates joint dysfunction and pain. Apoptotic cells also release damage-associated molecular patterns (DAMPs), which activate synovial macrophages, worsening the chronic inflammatory response and amplifying the pathological reactions of OA (Thomas et al., 2011; Zhou et al., 2019).

In conclusion, chondrocyte apoptosis plays an indispensable role in cartilage degeneration. By regulating chondrocyte apoptosis, it may be possible to alleviate cartilage degeneration and mitigate OA progression.

## 4 Key regulators of chondrocyte apoptosis

Many molecules are involved in regulating apoptosis, thereby affecting the progression of OA, such as caspases, P53, nitric oxide (NO), ROS, and non-coding RNAs (ncRNAs).

### 4.1 Caspase

Caspases are proteases found in the cytoplasm and play a crucial role in the process of cell apoptosis (Asadi et al., 2022). Typically, they exist as inactive zymogens that are cleaved and activated by proteolytic enzymes (Van Opdenbosch and Lamkanfi, 2019). Caspases are classified into two main categories: initiator caspases and executioner caspases. Initiator caspases include caspase-2, -8, -9, and -10, which are activated through interactions with the DED and caspase recruitment domain (CARD) of upstream proteins, triggering the caspase cascade (Yuan and Ofengeim, 2024; Alshiraihi and Kato, 2023). Executioner caspases, such as caspase-3, -6, and -7, are activated following cleavage by upstream initiator caspases and are responsible for degrading intracellular structural and functional proteins (Sahoo et al., 2023; Kesavardhana et al., 2020). Caspases-2, -8, and -10 are typically involved in death receptor-mediated apoptosis, while caspase-9 does not require removal of the N-terminal propeptide for activation. Caspase-9 forms the apoptosome with cytochrome C and APAF-1, playing a key role in mitochondrial apoptosis (Seo and Rhee, 2018). Studies have shown that small glutamine-rich tetratricopeptide repeat (TPR)-containing β and peroxisome proliferator-activated receptor gamma (PPARγ) protect cartilage by inhibiting the expression of caspase-3 (Bao et al., 2016; Yuan et al., 2024). Additionally, miR-146a-5p and miR-186-5p can prevent chondrocyte apoptosis by inhibiting caspase levels (Zhang, et al., 2021; Li Q. et al., 2021). Moreover, the release of Ca^2+^ from the ER can trigger the caspase cascade. Therefore, caspases are central to the three major apoptotic pathways: the death receptor pathway, the mitochondrial pathway, and the ER stress pathway.

### 4.2 P53

Studies have found that overexpression of p53 induces chondrocyte apoptosis (Qiu et al., 2024), which is associated with the upregulation of p53-upregulated modulator of apoptosis (P53A), further disrupting mitochondrial function in chondrocytes and promoting apoptosis. Studies have found that interventions with quinolone ofloxacin and uridine corticosterone upregulate p53 expression and induce chondrocyte apoptosis (Sheng et al., 2008; Lawrence et al., 2017), while drugs such as tripterygium (Lin et al., 2009), warm sparse-dense wave (Lin et al., 2017), and cytochalasin D (Kim, et al., 2003) can inhibit p53-mediated chondrocyte apoptosis. Additionally, external stimuli (such as shear stress) can also upregulate p53 levels, thereby promoting apoptosis (Hashimoto et al., 2009). The deficiency of Sirt1 leads to overexpression of p53, which triggers chondrocyte apoptosis (Xu et al., 2020), while Vam3 can inhibit chondrocyte apoptosis through Sirt1-mediated deacetylation of p53 (Jiang et al., 2014). Studies have found that microRNAs (miRNAs) can also regulate the expression of p53 and affect apoptosis. Overexpression of miR-363-3p increases p53 expression (Zhang et al., 2020a), while overexpression of miR-138 inhibits p53-mediated chondrocyte apoptosis (Xu et al., 2019) It is evident that p53 is a key regulatory factor in chondrocyte apoptosis.

### 4.3 NO and ROS

NO and ROS play important roles in the occurrence and development of OA. Studies have found that the level of NO is significantly increased in OA patients, while the level of ROS is decreased (Chen M. et al., 2021). NO induces apoptosis through the mitochondrial pathway by increasing the expression of pro-inflammatory factors (Grogan and D'Lima, 2010). Chemical donors of NO, such as sodium nitroprusside (SNP), can induce apoptosis in human and rabbit chondrocytes by increasing the expression of caspase-3 and -7 and inhibiting Bcl-2 levels. In addition, SNP can induce chondrocyte apoptosis by activating JNK, Bax translocation, mitochondrial dysfunction, and caspase activation (Mobasheri et al., 2015; Cheng et al., 2008). The effect of NO may be blocked by ROS. In the presence of NO, low concentrations of ROS induce apoptosis, while high concentrations of ROS lead to necrosis. Thus, the balance between NO and ROS determines the type of cell death in chondrocytes (Akkewar et al., 2024). Mitochondria are the primary source of ROS. During apoptosis, ROS damage the mitochondrial membrane structure, leading to a decrease in mitochondrial membrane potential and the release of apoptotic factors, which further promote ROS production and mitochondrial damage (Videla et al., 2022). This creates a vicious cycle, continuously promoting chondrocyte apoptosis. Research has shown that astragalus polysaccharides can reduce ROS levels *in vitro*, protecting chondrocytes from apoptosis. *In vivo*, they reduce chondrocyte apoptosis and alleviate cartilage degeneration via the ASK1/p38 signaling pathway (Xu et al., 2024). Angelica polysaccharides can reduce ROS levels in rat chondrocytes treated with tert-butyl hydroperoxide, improve mitochondrial function, enhance chondrocyte viability, and reduce chondrocyte apoptosis (Ni et al., 2023).

### 4.4 ncRNA

In recent years, it has been discovered that ncRNAs, including long noncoding RNAs (lncRNAs), miRNAs, and circular RNAs (circRNAs) (Matsui and Corey, 2017), which do not code for proteins, play important roles in regulating chondrocyte apoptosis. Numerous lncRNAs regulate apoptosis in OA at both the epigenetic and post-transcriptional levels. For example, lncRNA GAS5 may inhibit lipopolysaccharide (LPS)-induced chondrocyte apoptosis by activating miR-146a and miR-137 and upregulating Smad4 (Zhang and Qiu, 2021; Gao et al., 2020). LncRNAs such as CALML3-AS1, PACER, SNHG9, and RMRP inhibit chondrocyte apoptosis by regulating miRNAs(Nie et al., 2021; Jiang et al., 2019; Zhang et al., 2020b; Lu et al., 2020).

Furthermore, miRNAs are involved in OA by regulating cell apoptosis. The study of miR-140 has become one of the most prominent areas of research (Swingler et al., 2019). miR-140-5p is particularly important in OA development, while miR-140-3p, which is abundant in cartilage, plays a key role in regulating joint tissue homeostasis (Barter et al., 2015; Karlsen et al., 2014). Studies have shown that the level of miR-140 is lower in OA patients, and complete elimination of miR-140 in mice leads to impaired chondrocyte differentiation and proliferation (Nakamura et al., 2011). In IL-1β-treated chondrocytes, several miRNAs regulate apoptosis in various ways. Overexpression of miR-296-5p inhibits the expression of Bax/Bcl-2 and Caspase-9 (Cao et al., 2020). Inhibition of miR-126 suppresses the MAPK/JNK signaling pathway and upregulates Bcl-2 expression (Yu et al., 2018), while miR-29a targets Bax to inhibit chondrocyte apoptosis (Miao et al., 2019). In LPS-induced chondrocytes, miR-203a-3p and miR-93-5p inhibit chondrocyte apoptosis by acting on MYD88/NF-κB and lncRNA CASC2, respectively, protecting cartilage from damage (Chen et al., 2023; Sun et al., 2020).At the same time, circRNAs also participate in chondrocyte apoptosis. For example, overexpression of circCCDC66 and circZNF652 promotes chondrocyte apoptosis and increases the levels of IL-6 and TNF-α in chondrocytes (Zhang et al., 2022a; Yuan et al., 2021). CircRNA-9119 reduces IL-1β-induced chondrocyte apoptosis through the miRNA-26a/PTEN pathway (Chen et al., 2020), while circANKRD36 prevents OA chondrocyte apoptosis and inflammation by targeting miR-599 (Zhou et al., 2021).

It is evident that ncRNAs can serve as potential therapeutic targets for OA, which may aid in the development of appropriate drugs for treating the condition. However, clinical evidence is still needed to confirm the exact therapeutic targets for OA treatment.

## 5 Drugs for treating OA via apoptosis modulation

Currently, drugs for OA treatment primarily focus on alleviating symptoms such as pain and swelling, rather than preventing, delaying, or curing the disease. Since chondrocyte apoptosis plays a crucial role in cartilage degeneration, targeting the regulation of chondrocyte apoptosis may offer an effective strategy. Among the most researched apoptosis inhibitors for OA are caspase inhibitors. Caspase inhibitors such as Z-VAD-FMK, Z-DEVE-FMK (caspase-3 selective inhibitor), and Z-YVAD (caspase-1 selective inhibitor) have been shown to inhibit TNF-α, collagenase, and astroglycins-induced chondrocyte apoptosis *in vitro* (Yuan et al., 2024; Lo and Kim, 2004; Nuttall et al., 2000). Additionally, Z-DEVE-FMK and Z-LEHD-FMK (caspase-9 selective inhibitors) have been found to inhibit chondrocyte apoptosis in OA dogs *in vivo* (Pelletier et al., 2001). In a rabbit OA model established by anterior cruciate ligament transection (ACLT), Z-VAD-FMK reduced caspase-3 expression, inhibited apoptosis, and the combined use of caspase-1 and caspase-3 inhibitors reduced the pathological changes associated with OA (D'Lima et al., 2006). Shown in Table 1.

**TABLE 1:** Inhibitors of apoptosis targeting OA.

Type	Drug name	Model/Cell types	Route of treatment	Dosage	Duration	Signal pathways/ Mechanisms	Refs
Aspase inhibitor	Z-VAD-FMK	*In vitro*, human chondrocytes	cell seeding	10 μM	16 h	Inhibit ECM degradation	107
		*In vivo*, ACLT surgery rabbit	intra-articular injection	25 μg/mL	*In vivo*, 9 weeks	caspase-3	111
	Z-DEVE-FMK	*In vitro*, human chondrocytes	cell seeding	10 μM	16 h	Inhibit ECM degradation	108–109
	Z-LEHD-FMK	*In vitro*, canine chondrocytes	cell seeding	15 μM	24 h	P38/MERK1/2	110

Chondroprotective agents, including glucosamine sulfate, chondroitin sulfate, and hyaluronic acid, are widely used in the treatment of OA. Studies have shown that glucosamine hydrochloride/sulfate can reduce cartilage damage in OA models (both rabbits and humans), inhibit the expression of caspase-3 and caspase-9, increase Bcl-2 expression, and regulate the NF-κB pathway, thereby inhibiting chondrocyte apoptosis (Cheleschi et al., 2021; Chen et al., 2022; Jeong et al., 2018). Similarly, chondroitin sulfate has been shown to improve cell survival, reduce the apoptosis rate, and protect mitochondria in hydrogen peroxide-induced chondrocytes (Liu, et al., 2019). Hyaluronic acid injections can also inhibit chondrocyte apoptosis and protect cartilage tissue in both human and animal models (Wang et al., 2020; Barreto et al., 2015). Shown in Table 2.

**TABLE 2:** Chondroprotective agents for the treatment of OA.

Type	Drug name	Sources	Model/Cell types	Route of treatment	Dosage	Duration	Signal pathways/ Mechanisms	Refs
Chondroprotectant	Glucosamine Sulfate	—	*In vitro*, human chondrocytes	cell seeding	9 μM	24 h	NF-κB	112
			*In vivo*, ACLT surgery rabbit	oral administration	80 mg/kg	4 weeks	TRPV 5	113
			*In vivo*, ACLT surgery rabbit	oral administration	10 mg/kg	8 weeks	—	114
	Chondroitin sulfate	Sturgeon bone	*In vitro*, H_2_O_2_-induced rat chondrocytes	cell seeding	200, 400 μg/mL	12 h	caspase-3/caspase-9	115
	hyaluronic acid	—	*In vitro*, TNF-α-induced human chondrocytes *In vivo*, OA patients	*In vitro*, cell seeding *In vivo*, intra-articular injection	*In vitro*, 10 μM *In vivo*, 10 mg/mL	*In vitro*, 24 h *In vivo*, 2 weeks	AKT/NF-κB	116
			*In vivo*, impact-induced rabbit	intra-articular injection	10 mg/mL	30 days	—	117

The role of naturally sourced compounds in inhibiting chondrocyte apoptosis has gained significant attention. Compounds such as sulforaphane (Chen Q. et al., 2021), icariin (Huang et al., 2019), vitamin B6 (Fang et al., 2024), peach leaf coral glycoside (Wang et al., 2019), baicalin (Li et al., 2020), total saponins of Panax notoginseng (Zhang et al., 2022b), quercetin (Li W. et al., 2021), rhodioloside (Wu et al., 2019), andrographolide (Yan et al., 2022), curcumin (Li et al., 2017), geniposide (Pan et al., 2018), and oroxylin A (Wang et al., 2015) have been found to inhibit chondrocyte apoptosis by increasing the expression of anti-apoptotic proteins like Bcl-2 and suppressing pro-apoptotic proteins such as Bax and caspase-3 or caspase-9. Additionally, icariin, baicalin, quercetin, and oroxylin A can inhibit chondrocyte apoptosis via the mitochondrial pathway by reducing NO and ROS levels, inhibiting cytochrome C release, and maintaining mitochondrial membrane potential (Mi et al., 2018; Zhang et al., 2014; Hu et al., 2019). Furthermore, plant-derived compounds can act as inhibitors of ER stress, playing a significant role in protecting chondrocytes. For example, curcumin inhibits oxidative stress-induced chondrocyte apoptosis by promoting the expression of SIRT1 and suppressing the PERK/eIF-2α/ATF4/CHOP signaling pathway (Feng et al., 2019a).

Clinical studies have shown that baicalin’s chondroprotective effect on human OA chondrocytes is related to the inhibition of ER stress, as evidenced by the suppression of ER stress markers (BiP and CHOP) in hydrogen peroxide-pretreated chondrocytes (Cao et al., 2018). Additionally, trehalose inhibits oxidative stress-induced ER stress by reducing the levels of GRP78 and CHOP in tert-butyl hydroperoxide (TBHP)-treated chondrocytes (Chen et al., 2016). Quercetin inhibits ER stress primarily by regulating the signaling pathways of CHOP, p-PERK/PERK, p-IRE1α, and ATF6 (Feng et al., 2019b). Shown in Table 3.

**TABLE 3:** Natural compounds targeting chondrocyte apoptosis.

Type	Drug name	Sources	Model/Cell types	Route of treatment	Dosage	Duration	Signal pathways/ Mechanisms	Refs
Phytochemical	Sulforaphane	cruciferous vegetables	*In vivo*, H_2_O_2_-induced mice chondrocytes	cell seeding	50 µM	24 h	SIRT1	118
	Icariin	Epimedium	*In vivo*, collagenase-induced rat	oral administration	10, 20, 40 ng/mL	7 days	TDP-43	119
			*In vitro*, TNF-α-induced rat chondrocytes	cell seeding	10 μM	24 h	NF-κB	130
	Vitamin B6	—	*In vivo*, Collagen-induced mice	oral administration	40 mg/kg	4 weeks	Regulation of ECM metabolism	120
	Aucubin	*Eucommia ulmoides*	*In vitro*, IL-1β-induced mice chondrocytes *In vivo*, DMM surgery mice	*In vitro*, cell seeding *In vivo*, oral administration	*In vitro*, 50 μM *In vivo*, 50 mg/kg	*In vitro*, 24 h *In vivo*, 8 weeks	Inhibit excessive ROS production	121
	Baicalin	*Scutellaria baicalensis Georgi*	*In vitro*, IL-1β-induced human chondrocytes	cell seeding	20 μM	12 h	miR-766-3p/AIFM1	122
			*In vitro*, IL-1β and TNF-α-induced human chondrocytes	cell seeding	20, 50 μM	24 h	Inhibit ON production	131
			*In vitro*, H_2_O_2_-induced human chondrocytes	cell seeding	100 μM	24 h	Inhibit oxidative stress and endoplasmic reticulum stress	134
	*Panax notoginseng*	*notoginseng*	*In vitro*, TNF-α-induced rat chondrocytes *In vivo*, ACLT surgery rat	*In vitro*, cell seeding *In vivo*, intra-articular injection	*In vitro*, 100, 200 μg/mL *In vivo*, 75 μmol/L	*In vitro*, 24 h *In vivo*, 8 weeks	PI3K/AKT/mTOR	123
	Quercetin	Many fruits and vegetables	*In vitro*, IL-1β-induced rat chondrocytes *In vivo*, ACLT surgery rat	*In vitro*, cell seeding *In vivo*, intra-peritoneal injection	*In vitro*, 8 μM *In vivo*, 50 mg/kg	*In vitro*, 48 h *In vivo*, 12 weeks	IRAK1/NLRP3	124
			*In vitro*, IL-1β-induced rat chondrocytes *In vivo*, DMM surgery rat	*In vitro*, cell seeding *In vivo*, intra-articular injection	*In vitro*, 8 μM *In vivo*, 8 μM	*In vitro*, 24 h *In vivo*, 6 weeks	Regulating the polarization of synovial macrophages to M2 macrophages	132
			*In vitro*, IL-1β-induced rat chondrocytes *In vivo*, DMM surgery rat	*In vitro*, cell seeding *In vivo*, intra-peritoneal injection	*In vitro*, 25 μM *In vivo*, 50 mg/kg	*In vitro*, 24 h *In vivo*, 12 weeks	SIRT1/AMPK	135
	Salidroside	*Rhodiola rosea*	*In vitro*, IL-1β-induced rat chondrocytes	cell seeding	100 μmol/L	24 h	PI3K/AKT	125
	Andrographolide	*Andrographis paniculata*	*In vitro*, LPS-induced mice chondrocytes *In vivo*, ACLT surgery mice	*In vitro*, cell seeding *In vivo*, intra-articular injection	*In vitro*, 25 μM *In vivo*, 50 mg/kg	*In vitro*, 12 h *In vivo*, 3 weeks	Circ-Rapgef1/miR-383-3p/NLRP3	126
	Curcumin	*Curcuma longa*	*In vitro*, IL-1β-induced rat chondrocytes	cell seeding	10 μM	24 h	ERK 1/2	127
			*In vitro*, TBHP-induced rat chondrocytes *In vivo*, ACLT surgery rat	*In vitro*, cell seeding *In vivo*, oral administration	*In vitro*, 20 μM *In vivo*, 50, 150 mg/kg	*In vitro*, 24 h *In vivo*, 8 weeks	PERK/eIF2α/CHOP	133
	Geniposide	*Gardenia jasminoides*	*In vitro*, IL-1β-induced rat chondrocytes *In vivo*, MMT surgery rat	*In vitro*, cell seeding *In vivo*, intra-peritoneal injection	*In vitro*, 2.5、5.0, 10 μM *In vivo*, 10 mg/kg	*In vitro*, 24 h *In vivo*, 8 weeks	ePI3K/Akt/NF-κB	128
	Shikonin	Arnebia euchroma	*In vitro*, IL-1β-induced rat chondrocytes	cell seeding	4 μM	24 h	PI3K/AKT	129

## 6 Challenges in translating apoptosis research into OA therapeutic drugs

Z-VAD-FMK, a broad-spectrum caspase inhibitor, rapidly inhibits most caspases *in vitro*. However, it is not suitable as a therapeutic lead due to the production of toxic fluoroacetate (Poreba et al., 2013). The caspase-1 peptide mimic inhibitor VX-740 (pralnacasan) has shown promising efficacy in clinical trials for OA treatment (Poreba et al., 2013; Rudolphi et al., 2003). Unfortunately, these trials were terminated due to liver toxicity observed in long-term animal studies (Dhani et al., 2021). Despite the potential of caspase inhibitors in OA treatment, few drugs have advanced to clinical trials, primarily because of discrepancies between animal models and human clinical trial protocols (Dhani et al., 2021).

UBX-0101 (Unity Biotechnology Inc., structure undisclosed) is another compound that inhibits the binding of p53 to MDM2, promoting apoptosis in senescent chondrocytes. In a Phase I clinical trial (NCT03513016) involving 48 subjects, intra-articular injection of UBX-0101 at doses of 1–4 mg effectively relieved OA-related pain with no significant side effects. However, in a Phase II trial (NCT04129944) involving 180 patients, UBX-0101 failed to meet the primary clinical endpoints, leading to the termination of further research (Zhang et al., 2024).

Thus, translating apoptosis research into OA treatments faces several challenges, including: (1) Unclear efficacy evaluation standards: OA drugs must demonstrate both cartilage structure improvement and symptom relief. Validating cartilage structure improvement, however, requires long-term follow-up, which is costly and resource-intensive. (2) Patient heterogeneity: OA has various etiologies, and apoptosis may play a dominant role in specific subgroups. This necessitates precise patient stratification to increase the success rate of clinical trials. (3) Safety risks: Long-term use of apoptosis inhibitors may increase the risk of tumors or interfere with normal tissue regeneration, potentially leading to other diseases.

In the future, translating apoptosis research into clinical OA treatments will require interdisciplinary collaboration to address key issues such as target specificity, delivery efficiency, and clinical validation. Overcoming these challenges will be essential for transforming mechanistic research into successful clinical applications.

## 7 Discussion

As the global aging population continues to rise, the number of OA patients is expected to increase, significantly impacting the quality of life for patients and placing a growing burden on society (Wu et al., 2025). Current drug treatments for OA mainly focus on alleviating symptoms, such as pain and swelling, but these therapies often come with side effects. Therefore, the search for effective drugs to delay the progression of OA holds immense clinical and practical value.

In the early stages of OA, moderate apoptosis can help remove damaged cells and slow the spread of inflammation. However, excessive apoptosis triggers oxidative stress and inflammatory responses, leading to abnormal ECM secretion, cartilage degradation, and an acceleration of disease progression. In the later stages of OA, the destruction of cartilage structure and the subsequent production of pro-inflammatory factors further exacerbate cartilage damage, making it a primary pathological phenomenon. This highlights that apoptosis plays a dual role in OA development, with both protective and detrimental effects. Targeting chondrocyte apoptosis could, therefore, be an effective therapeutic strategy to delay cartilage degeneration.

During the early stages of OA, inducing apoptosis may help target and remove damaged, aging chondrocytes, thereby restoring the cartilage tissue microenvironment and potentially preventing further cartilage degeneration. On the other hand, inhibiting excessive apoptosis and protecting chondrocytes as early as possible is also a valuable therapeutic approach. One promising strategy involves targeting the ER stress pathway. ER stress, triggered by various exogenous stimuli, contributes to cartilage degradation. Inhibiting ER stress could block several pathogenic factors (Yang et al., 2025), and since ER stress is an early stage of chondrocyte apoptosis, effectively suppressing it could protect cartilage tissue and delay degeneration (Yu et al., 2025). Natural products, known for their high activity, low side effects, and broad availability, hold significant research potential. Studies have shown that natural compounds can protect articular cartilage by inhibiting chondrocyte apoptosis in both *in vivo* and *in vitro* models. Consequently, natural drugs that target chondrocyte apoptosis inhibition have great prospects for development and may become new clinical treatment options in the future (Yu et al., 2025; Kong et al., 2022; Posey, 2024). However, current research is still largely at the animal or cellular level, and further challenging basic research is necessary to advance the understanding of their safety and efficacy in clinical settings.

At the same time, there are still many issues regarding the scientific mechanisms for regulating apoptosis in the prevention and treatment of OA. First, the boundary between protective and pathological apoptosis is blurred, and there is a lack of clear biomarkers to distinguish the different forms of apoptosis, which could result in treatment strategies causing harm to normal cells. Secondly, there is insufficient spatiotemporal specificity in apoptosis regulation. The role of apoptosis may differ at different stages of OA and in different tissues, making it difficult to precisely target chondrocyte apoptosis interventions. This could cause side effects in synovial or bone tissues, with insufficient research evidence in this area. Furthermore, apoptosis intersects with other forms of cell death. In OA, apoptosis overlaps with necroptosis, pyroptosis, and autophagy, and solely targeting apoptosis regulation may not fully block the pathological progression. There are also technical bottlenecks that need to be overcome. First, the limitation of targeted delivery systems. Most apoptosis-targeting drugs are administered systemically, making it difficult to precisely target the joint area, which may lead to systemic toxicity. Additionally, the delivery efficiency and targeting ability of nanoparticle carriers or gene editing technologies in joints need optimization and industrialization. Secondly, most studies rely on mouse or rat OA models, but their pathological progression differs significantly from that in humans. This makes it difficult to simulate the chronic progression and heterogeneity of OA, leading to preclinical results that are hard to replicate in humans.

There are still many shortcomings in both basic research and clinical translational research. In basic research, it is recognized that different subtypes of OA—such as inflammation-dominant, mechanical stress-dominant, and metabolic abnormality-dominant types—may respond differently to apoptosis interventions. However, the mechanistic studies addressing these differences have not been fully developed. Additionally, there is a lack of in-depth research on the interactions between epigenetics and the microenvironment. The role of epigenetic modifications, such as DNA methylation or histone acetylation, and environmental factors like hypoxia and mechanical stress in influencing apoptosis regulation and related signaling pathways remains underexplored. In clinical translational research, several challenges persist. First, there is a lack of stage-specific clinical trials. Many intervention studies fail to distinguish between early and late-stage OA patients, leading to confounded efficacy results. For instance, drugs that inhibit apoptosis may show little effectiveness in late-stage OA but could be beneficial in early-stage subgroups. Second, there is uncertainty about the long-term safety and efficacy of apoptosis-modulating drugs. Intervening in apoptosis regulation could disrupt normal physiological processes, such as immune surveillance or tissue homeostasis, raising concerns that prolonged use may increase the risk of infections or degenerative diseases.

## 8 Conclusion

In conclusion, this review highlights the relationship between chondrocyte apoptosis and cartilage degeneration, emphasizing that targeting chondrocyte apoptosis presents a promising approach for treating cartilage degradation in OA. The review also outlines the key factors regulating chondrocyte apoptosis and discusses various drugs that target apoptosis, offering valuable insights for future therapeutic and mechanistic studies on OA.

However, the translation of mechanistic findings into clinical applications remains a challenge and requires further exploration in the following areas: (1) Development of spatiotemporal-specific regulatory tools: Utilizing advanced techniques such as conditional gene knockout, overexpression systems, or light- and temperature-controlled drugs to achieve local, precise regulation of apoptosis. (2) Research on multi-tissue interactions: Subchondral bone, located beneath the articular cartilage, plays an active role in the pathogenesis of OA. Future studies should focus on the interactions between cartilage and subchondral bone in apoptosis to uncover the broader pathological mechanisms of OA. (3) Exploring multimodal combined treatment strategies: Combining apoptosis regulation with anti-inflammatory, antioxidant, or matrix-synthesis-promoting agents could synergistically improve the OA microenvironment and enhance therapeutic outcomes. (4) Optimization of clinical trial design: Patient stratification using biomarkers (e.g., Caspase-3 fragments in serum or cartilage oligomeric matrix protein) and designing stage- and subtype-specific clinical trials will improve the precision and effectiveness of therapeutic interventions. (5) Application of novel model systems: Employing organoid or humanized OA models to improve the clinical relevance of mechanistic research and drug screening processes. (6) Development of natural product apoptosis-targeted formulations: Natural products can be modified with nanomaterials or polymers to actively or passively target disease-related sites, enabling more precise therapeutic effects (Zhang et al., 2024; Zhang and Yang, 2024). Additionally, novel excipients, such as pressure-sensitive adhesives, can be used to formulate transdermal topical applications for local drug delivery to the joints. This approach aims to reduce dosage frequency, improve patient safety, and enhance adherence (Song et al., 2024). Furthermore, these formulations can exert systemic effects by being absorbed into the bloodstream, bypassing hepatic first-pass metabolism, and thereby promoting the broader utilization and development of natural compounds.

## Abbreviations

OA, osteoarthritis; ECM, extracellular matrix; APAF1, apoptotic protease activating factor-1; FADD, Fas-associating protein with a novel death domain; DED, death effector domain; DD, death domain; CARD, caspase recruitment domain; DISC, death-inducing signaling complex; MOMP, mitochondrial membrane potential permeability; XIAP, X-linked inhibitor of apoptosis protein; Smac, second mitochondria-derived activator of caspases; ILs, interleukins; TNFs, tumor necrosis factors; PERK, protein kinase R-like endoplasmic reticulum kinase; IRE1, inosital-requiring enzyme-1; ATF6, activating transcription factor 6; eIF-2α, eukaryotic translation initiation factor 2 alpha; CHOP, C/EBP-homologous protein; GADD153, growth arrest and DNA damage-inducible 153; TRAF-2, receptor-associated Factor-2; ROS, reactive oxygen species; NO, nitric oxide; MMPs, matrix metalloproteinases; SNP, sodium nitroprusside; TBHP, tert-butyl hydroperoxide; lncRNAs, long noncoding RNAs; miRNAs, microRNAs; circRNAs, circular RNAs.
